# Does the Oral Microbiome Play a Role in Hypertensive Pregnancies?

**DOI:** 10.3389/fcimb.2020.00389

**Published:** 2020-07-30

**Authors:** Thomas Willmott, Andrew J. McBain, Gavin J. Humphreys, Jenny Myers, Elizabeth Cottrell

**Affiliations:** ^1^Maternal and Fetal Health Research Centre, Division of Developmental Biology & Medicine, School of Medical Sciences, Faculty of Biology, Medicine and Health, The University of Manchester, Manchester, United Kingdom; ^2^Division of Pharmacy and Optometry, School of Health Sciences, Faculty of Biology, Medicine and Health, The University of Manchester, Manchester, United Kingdom

**Keywords:** oral microbiota, nitrite, dietary nitrate, nitric oxide, blood pressure, hypertension, beetroot juice, pregnancy

## Abstract

Chronic hypertension during gestation is associated with an increased risk of adverse pregnancy outcomes including pre-eclampsia, fetal growth restriction and preterm birth. Research into new chemotherapeutic regimes for the treatment of hypertension in pregnancy is limited due to concerns about fetal toxicity and teratogenicity, and new therapeutic avenues are being sought in alternative physiological pathways. Historically, generation of the vasodilator nitric oxide was believed to be solely from *L*-arginine by means of nitric oxide synthase enzymes. Recently, a novel pathway for the reduction of dietary inorganic nitrate to nitrite by the bacteria in the oral cavity and subsequently to vasodilatory nitric oxide within the body has been uncovered. Dietary nitrate is abundant in green leafy vegetables, including beetroot and spinach, and reduction of exogenous nitrate to nitrite by oral bacteria can increase nitric oxide in the vasculature, lessening hypertension. Supplements rich in nitrate may be an attractive choice for treatment due to fewer side effects than drugs that are currently used to treat hypertensive pregnancy disorders. Additionally, manipulation of the composition of the oral microbiota using pro- and prebiotics in tandem with additional dietary interventions to promote cardiovascular health during gestation may offer a safe and effective means of treating hypertensive pregnancy disorders including gestational hypertension and pre-eclampsia. The use of dietary inorganic nitrate as a supplement during pregnancy requires further exploration and large scale studies before it may be considered as part of a treatment regime. The aim of this article is to review the current evidence that oral microbiota plays a role in hypertensive pregnancies and whether it could be manipulated to improve patient outcomes.

## Introduction

Hypertension is a modern medical crisis in all nations across the globe due to its correlation with a poor, high calorie diet and minimal exercise, all of which are risk factors for cardiovascular disease and death (Magee et al., 2014). In pregnancy, hypertensive disorders increase the risk of an adverse pregnancy outcome, with complications for mother and child, including pre-eclampsia (PE), fetal growth restriction (FGR) and pre-term birth (Bramham et al., 2014; Moussa et al., 2014; Webster et al., 2019). Hypertensive pregnancy disorders (HPDs) now complicate up to 10% of all pregnancies and rates are expected to continue to rise (Firoz et al., 2014; Myers, 2017). In addition to perinatal risks, HPDs carry maternal risk into the post-partum period, with increased rates of coronary artery disease, ischemic heart disease, stroke, and other cardiovascular diseases compared with women who remained normotensive throughout their pregnancies (Bellamy et al., 2007; McDonald et al., 2008). Due to concerns about neonatal teratogenicity there is resistance from pharmaceutical companies to develop new anti-hypertensive medicines in addition to a reluctance for clinical intervention from both clinicians and mothers (Bahadoran et al., 2018; Excellence NIfGaC). It therefore remains a priority to develop alternative, novel avenues for therapeutic intervention.

## Nitric Oxide and Cardiovascular Health

Nitric oxide (NO) is a vital signaling molecule in the cardiovascular system and has been implicated in numerous vascular processes including vasodilation and tissue protection (Lundberg et al., 2008). The generation of NO by metabolism of *L*-arginine via host nitric oxide synthases (NOS) is crucial in maintaining vascular homeostasis within the host (Cannon, 1998) and limited bioavailability of NO has been demonstrated in chronic cardiovascular disorders including hypertension (Panza et al., 1990) and hypercholesterolemia (Chowienczyk et al., 1992). NO regulates vascular tone (and hence blood flow) by activation of soluble guanylate cyclase (sGC) in the smooth muscle cells (Luiking et al., 2010), increasing the production of 3′5′-cyclic guanosine monophosphate (cGMP), relaxing smooth muscle, and causing vessel vasodilation (Zhao et al., 2015). NO has a short biological half-life of 3–5 s (Zhao et al., 2015) and can be quickly scavenged or oxidized by hemoglobin to nitrite and eventually nitrate in the plasma (Biswas et al., 2014), attenuating its local effects.

In addition to endogenous synthesis, NO can be derived from exogenous sources; nitrogenous compounds can be introduced into the body through diet and drinking water. Green, leafy vegetables, such as spinach and lettuce, concentrate nitrate in their leaves, whilst other plants such as beetroot store nitrate in their swollen roots at extremely high concentrations, in the region of 1,500 mg/kg in beetroot (Tamme et al., 2006; Gilchrist et al., 2009). These nitrates are derived from bacterial nitrogen fixation and are utilized in plant protein production from amino acids using energy derived from photosynthesis (Gilchrist et al., 2009). Consumption of these vegetables form the largest proportion of overall nitrate intake in humans (Hobbs et al., 2013). In a review of the literature, Hobbs et al. (2013) summarized comparative nitrate content in common dietary vegetables and found that the high-nitrate-accumulating vegetables were Brassicaceae (rocket), Chenopodiaceae (beetroot and spinach), Asteraceae (lettuce), and Apiaceae (celery).

**Figure 1:**
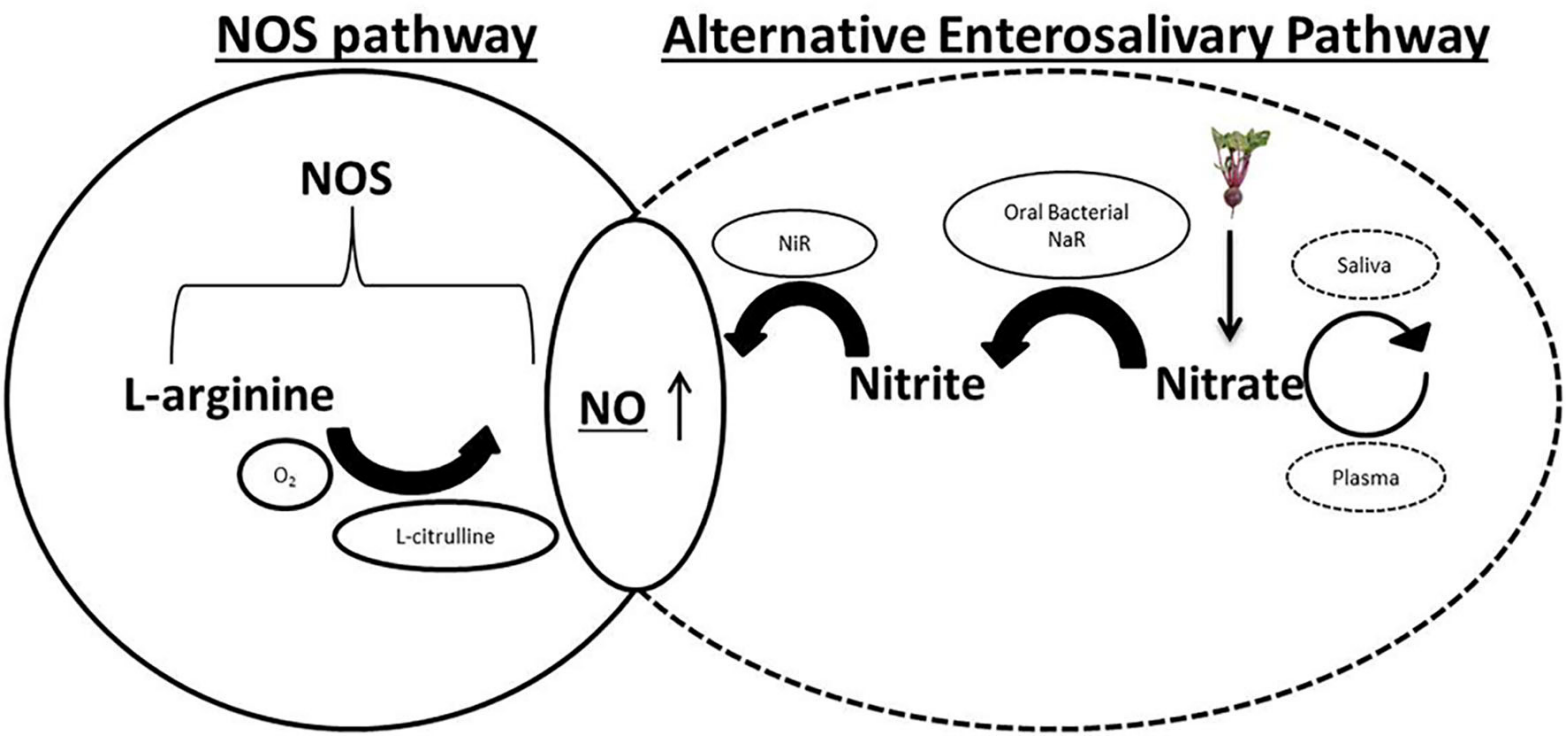
Physiological pathways of nitric oxide (NO) generation. Endogenous *L-*arginine is oxidized by the nitric oxide synthases (NOS) enzymes to NO and *L-*citrulline in the traditional NOS pathway. In the alternative enterosalivary pathway, dietary nitrate is absorbed across the gastrointestinal (GI) epithelium into the bloodstream before being actively concentrated in the salivary glands by sialic acid transporters. Reduction by oral bacterial species possessing nitrate reductase enzymes (NaR) generates nitrite, which enters the GI tract, is reabsorbed into the plasma and reduced to NO by bacterial and host nitrite reductases (NiR).

Following ingestion, dietary nitrate is rapidly absorbed across the epithelium of the upper gastrointestinal tract (Hawksworth and Hill, 1971), into the plasma (Figure 1). It has been estimated that around two thirds of this nitrate is excreted into the urine following glomerular filtration in the kidneys (Green et al., 1981). Clinical studies have estimated plasma nitrate concentration during fasting to be between 20 and 40 μM (Webb et al., 2008). The oxidation of endogenous NO, originating from the metabolism of arginine by NOS enzymes, serves as a secondary source of nitrate ions in the plasma (Kapil et al., 2014) and mixes with the diet-derived nitrate. Plasma nitrate is then concentrated in the salivary glands by sialic acid transporters (Tannenbaum et al., 1976; Qin et al., 2012). This is the mechanism by which high concentrations of nitrate are introduced into the oral cavity, in which the oral bacteria play the next part of *in vivo* recycling of nitrate to nitrite and subsequently NO in the human body. This is the alternative pathway for NO generation, discovered around 20 years ago (Duncan et al., 1995), in which dietary nitrate is reduced to nitrite by the oral microbes and further to NO in the bloodstream and tissues (Lundberg et al., 2004, 2018). This pathway had previously been largely ignored as a determinant of NO homeostasis in cardiovascular health and disease. However, there is now an emerging field of research investigating the role of microbiome driven NO production for the treatment of hypertensive disorders (Bryan et al., 2017).

## Oral Microbiota and the Enterosalivary Pathway

Prokaryotes, unlike mammalian cells, lack the NOS enzymes that catalyze the production of NO from *L*-arginine (Qu et al., 2016). Host microbes rely on dietary nitrates which are swallowed and rapidly absorbed across the epithelium in the GI tract as a source of nitrogen. Nitrate is actively concentrated in the salivary glands after being removed from the plasma and once the saliva is secreted into the mouth, the nitrate is reduced by select oral bacterium to nitrite, utilizing their nitrate reductase enzymes. The surface of the tongue has been determined to be the site of the majority of the nitrate reducing bacteria (Doel et al., 2005). Following swallowing, nitrite can be converted into nitric acid in the low stomach pH by protonation or further reduced to NO by bacterial nitrite reductase enzymes in the gastrointestinal tract, where it has various roles including maintaining the gastric epithelium mucus barrier and mediating gastric blood flow (Lanas, 2008). These latter reactions can facilitate the production of nitro-fatty acids, such as NO_2_ conjugated linoleic acid. These compounds are introduced into the plasma, thus completing the pathway and consequently resulting in higher bioavailability of NO and other nitrogen oxides in the vasculature (Koch et al., 2017).

Using 16S rRNA gene pyrosequencing and whole genome shotgun sequencing, Hyde et al. (2014) reported that *Neisseria, Veillonella, Haemophilus, Porphyromonas, Fusobacterium, Prevotella, Leptorichia, Brevibacillus*, and *Granulicatella* contributed most significantly to nitrate reduction in the mouth. The authors examined the amplicons of tongue scrapings, running *in vitro* single- and polymicrobial biofilm nitrate reduction assays on isolates and sequencing of selected species, in addition to calculating the relative abundances of nitrate reductase genes following whole genome sequencing. Other studies have additionally linked the genera *Rothia* and *Staphylococcus* with oral nitrate reduction (Li et al., 1997; Doel et al., 2005). Burleigh et al. (2018) concluded that *Prevotella* was the most abundant genera of nitrate-reducing bacteria in the oral cavity, as determined by relative abundances of bacterium on the tongue surface, which have been previously implicated in nitrate reduction.

Disruption of the oral microbiota using mouthwash has been shown to correlate with a reduction in plasma and salivary nitrite (Woessner et al., 2016) and disturbing these oral commensals and subsequent cardiometabolic homoeostasis with antibacterial mouthwashes has been suggested as a major risk factor in the development of diabetes mellitus (Joshipura et al., 2017; Long et al., 2017). This has been further linked to an elevated blood pressure (BP) in hypertensive participants (Bondonno et al., 2015), yet this can be overcome by direct supplementation with oral nitrite (Pinheiro et al., 2016).

In terms of pregnancy, epidemiological studies have identified a positive correlation between periodontal disease and preeclampsia (Wei et al., 2013), as well as an association between maternal oral health and various adverse pregnancy and birth outcomes, early childhood caries, and other chronic diseases (Vamos et al., 2015). These findings indicate a complex and multi-faceted role of the oral microbiota in health and disease, including during pregnancy.

Taken together, these studies suggest that the oral microbiota is a key player in systemic cardiovascular health and may play a role in regulating BP, including in pregnancy, although the precise physiological mechanisms are unclear and more detailed investigations are required. Toward this end, we are currently conducting a clinical trial aimed at investigating the role of the oral microbiome in HPDs (study identifier: NCT03930693).

## Dietary Interventions for Hypertension

There is now abundant evidence that dietary nitrate supplementation, particularly from vegetables, can increase the bioavailability of NO in the vasculature to ultimately increase vasodilation and decrease BP (Webb et al., 2008; Hobbs et al., 2012; Kapil et al., 2015; Velmurugan et al., 2016). Supplementation with dietary nitrate has also been shown to increase blood flow *in vivo* both in animal models (Ferguson et al., 1985, 2013) and in humans (Casey et al., 2007; Wightman et al., 2015; Richards et al., 2018). For regulatory reasons, beetroot juice supplementation has been used most widely in these studies (Flueck et al., 2015). The high inorganic nitrate content of beetroot juice has been proposed to increase the bioavailability of NO (Bahadoran et al., 2017) and thus can be utilized to treat cardiovascular and metabolic disorders including hypertension (Coles and Clifton, 2012). Supplementation with beetroot juice in non-pregnant adults significantly lowers BP and improves vascular function, which has been demonstrated in numerous experimental studies (Webb et al., 2008; Kapil et al., 2010; Bondonno et al., 2012; Hobbs et al., 2012; Kelly et al., 2013). Conversely, some studies have reported no effects on BP from dietary nitrate dosing (Gilchrist et al., 2013; Bondonno et al., 2015; Ormesher et al., 2018), however, it is hypothesized that the variances in efficacy of nitrate supplementation between individuals is related to differences in the oral bacterial profiles (Burleigh et al., 2018; Ormesher et al., 2018; Zhurakivska et al., 2019).

## Dietary Interventions for Hypertensive Pregnancy Disorders

In terms of translating these findings into pregnancy research, beetroot juice is more attractive as an intervention due to it being potentially more acceptable than traditional anti-hypertensive chemotherapeutic medications (Fisk and Atun, 2008; Cottrell et al., 2017). Interestingly, ingestion of green leafy vegetables has been associated with improved pregnancy outcomes (Chappell et al., 2013; McCowan et al., 2017). In our small feasibility trial in which beetroot juice was given for 1 week during mid-pregnancy, 97% of participating women found this an acceptable intervention (Ormesher et al., 2018), suggesting that women with pregnancy complications would be willing to use such an intervention if it proved effective.

In contrast, there are some potential health concerns highlighted regarding prolonged exposure over several years to excess dietary nitrates through drinking water. For many years, nitrates were strictly controlled in public tap water and avoided in pregnancy, due to concern about their perceived mutagenic, teratogenic, and carcinogenic traits (Gilchrist et al., 2009). However, estimates of nitrate intakes by individuals adhering to the “Dietary Approaches to Stop Hypertension” (DASH) diet suggest that nitrate intakes would be ~550% higher compared with World Health Organization acceptable limits (Hord et al., 2009), questioning the relevance of these regulated limits. Secondly, other acute toxicities, namely methemoglobinemia, were a concern, with preliminary studies five decades ago observing that oral administration of nitrites [30 mg/kg dose of sodium nitrite (NaNO_2_) per oral administration; a high pharmacological dose] led to methemoglobinemia in a pregnant rat model (Gruener et al., 1973). Yet more recent experimental studies have reported no elevation of methemoglobinemia following a 6-day administration of NaNO_2_ in 12 patients with diabetes mellitus, with no increase in serum methemoglobin or any other safety concerns (Greenway et al., 2012). Other worries included the generation of peroxynitrite and reactive nitrogen intermediates (Bahadoran et al., 2015), although recent studies have disputed these claims following sodium nitrate supplementation (Larsen et al., 2011). There is the potential for the conversion of nitrate to N-nitroso compounds (NOC) through nitrozation (Bahadoran et al., 2018) and most NOC are considered to behave as carcinogens and teratogens (Ward et al., 2018). However again, the evidence that exposure to nitrates through drinking water is correlated with cancer is conflicting (Ward et al., 2018), with more research required to eliminate confounding contaminants and risk factors. Bahadoran et al. (2018) also suggested that both nitrate and nitrite may be actively transported across the placenta, potentially resulting in high levels in fetal plasma and tissues. Furthermore, nitrate is a sodium/iodide symporter inhibitor (Bahadoran et al., 2018), potentially affecting thyroid function through nitrate-induced iodide deficiency (Eskiocak et al., 2005). Some studies have even argued nitrite added as a preservative in cured meats could induce pediatric brain tumors (Ortega et al., 2001) and neural tube defects in the fetus (Croen, 2001), although evidence is extremely limited. Taking all the evidence together, the idea of using dietary nitrate as a therapeutic in pregnancy is still controversial, and there is much to be discussed, evaluated, and tested before it can be recommended for HPDs.

## The Potential of the Oral Microbiota to Regulate Blood Pressure in Pregnancy

Investigations from our research group have recently demonstrated that nitrate supplementation, from beetroot juice, in pregnant women was associated with a reduction in BP that correlated with the degree of plasma nitrite increase (Ormesher et al., 2018). The standardized beetroot juice, containing ~400 mg of nitrate, led to highly variable changes in plasma nitrite between women, suggesting that the oral conversion of nitrate to nitrite, mediated by the oral microbiota, might be of key importance in determining the efficacy of this dietary intervention. This hypothesis is supported by growing evidence that oral bacteria may be major determinates of healthy BP regulation, in addition to BP responses to nitrate dosing (Kapil et al., 2013; Velmurugan et al., 2016; Koch et al., 2017; Burleigh et al., 2019). Consequently, the exploration of the role of the oral microbiota in BP regulation is an exciting therapeutic avenue for the treatment of hypertension. Alterations in the composition of the oral microbiota may therefore be utilized as a biomarker for prediction of nitrate responses, and shifting the microbiota through chronic supplementation strategies or the development and use of new probiotics with a known high nitrate reducing capacity, might improve BP regulation. Alternative probiotics, potentially using Veillonella or Actinomyces species, will have to be investigated for use as current Lactobacilli and Bifidobacterium oral probiotics are not known high nitrate-reducing species, with Lactobacilli potentially playing an inhibitory role in oral nitrate reduction (Hyde et al., 2014). However, there are safety considerations associated with the use of probiotics that are not GRAS, such as Veillonella or Actinomyces. This will require thorough *in vitro* and *in vivo* studies for efficacy and safety before these probiotics could be considered for human use.

Research interest in the oral microbiota and its relationship with BP responses has become more prominent within the last few years (Bryan et al., 2017). The ability to shift the composition of the oral microbiota via short term dietary nitrate supplementation in a small population of healthy volunteers has recently been demonstrated (Koch et al., 2017). In response to 10 days of dietary nitrate supplementation, there was a marked increase in relative abundance of Proteobacteria (+225%) and a reduction in Bacteroidetes (−46%) (Vanhatalo et al., 2018). In a separate study, using a 7 day nitrate supplemented diet, increased vascular NO levels and reduced BP were observed in 11 healthy male participants (Burleigh et al., 2019). Further *in vitro* models have demonstrated a shift in the composition of oral microcosms (up to 5 linear discriminant analysis effect size score) toward higher relative abundances of *Neisseria* and *Veillonella* following nitrate supplementation (Koopman et al., 2016). Evidence in the literature now strongly suggests that the oral microbiome can be shifted to improve BP regulation via nitrate metabolism, and investigation with future studies is justified.

## Conclusion and Future Directions

Given the positive effects of nitrate supplementation in non-pregnant populations, and our preliminary findings in pregnant women, it remains possible that dietary interventions targeting the nitrate-nitrite-NO pathway could be used in future to improve outcomes in HPDs. Investigating whether differences in the oral microbiome are associated with HPDs, and determining the efficacy of BP responses to dietary nitrate supplementation amongst different groups of pregnant women, is the focus of our current studies. Future trials should explore and evaluate whether nitrate supplementation and/or probiotic supplementation can improve pregnancy outcomes in hypertensive pregnant women. Research should also be expanded to other maternal microbiotas which are known to be dramatically altered during gestation, such as the gut (Koren et al., 2012; Edwards et al., 2017), and with a greater focus on NO metabolism, to fully determine the possible effects of these proposed dietary interventions (Koch et al., 2017). If effective, this approach could significantly improve management of HPDs and therefore maternal and fetal outcomes. This is an exciting prospect for an area of biomedical science which is under-researched and under-funded.

## Author Contributions

All authors contributed to the writing of the manuscript and approved the final submitted version.

## Conflict of Interest

The authors declare that the research was conducted in the absence of any commercial or financial relationships that could be construed as a potential conflict of interest.
